# Phase II Trial of Adjuvant S-1 Following Neoadjuvant Chemotherapy and Surgery in Patients with Locally Advanced Esophageal Squamous Cell Carcinoma: The PIECE Trial

**DOI:** 10.1245/s10434-024-16325-2

**Published:** 2024-10-07

**Authors:** Motoo Nomura, Toshifumi Yamaguchi, Keisho Chin, Shinji Hato, Ken Kato, Eishi Baba, Hisahiro Matsubara, Hidenori Mukaida, Takako Yoshii, Masahiro Tsuda, Yasuhiro Tsubosa, Yuko Kitagawa, Isao Oze, Hideki Ishikawa, Manabu Muto

**Affiliations:** 1 Department of Clinical Oncology, Kyoto University Hospital, Kyoto, Japan; 2 Department of Head and Neck Oncology and Innovative Treatment, Graduate School of Medicine, Kyoto University, Kyoto, Japan; 3 Cancer Chemotherapy Center, Osaka Medical and Pharmaceutical University Hospital, Takatsuki, Japan; 4 Gastroenterological Chemotherapy Department, Cancer Institute Hospital of Japanese Foundation for Cancer Research, Koto-ku, Tokyo, Japan; 5 Department of Gastroenterological Surgery, National Hospital Organization Shikoku Cancer Center, Matsuyama, Japan; 6 Department of Head and Neck, Esophageal Medical Oncology, National Cancer Center Hospital, Chuo-ku, Tokyo, Japan; 7 Department of Comprehensive Oncology, Graduate School of Medical Sciences, Kyushu University, Fukuoka, Japan; 8 Department of Frontier Surgery, Graduated School of Medicine, Chiba University, Chiba, Japan; 9 Department of Surgery, Hiroshima City Asa Citizens Hospital, Hiroshima, Japan; 10 Department of Gastroenterology, Saitama Cancer Center, Kitaadachi-gun, Saitama, Japan; 11 Department of Gastroenterological Oncology, Hyogo Cancer Center, Akashi, Japan; 12 Division of Esophageal Surgery, Shizuoka Cancer Center Hospital, Sunto-gun, Shizuoka, Japan; 13 Department of Surgery, Keio University School of Medicine, Minato-ku, Tokyo, Japan; 14 Division of Cancer Information and Control, Aichi Cancer Center Research Institute, Nagoya, Japan; 15 Department of Molecular-Targeting Prevention, Kyoto Prefectural University of Medicine, Kyoto, Japan; 16 Department of Clinical Oncology, Graduate School of Medicine, Kyoto University, Kyoto, Japan

**Keywords:** Esophageal squamous cell carcinoma, Neoadjuvant chemotherapy, Surgery, Adjuvant chemotherapy, S-1

## Abstract

**Background:**

Neoadjuvant chemotherapy followed by surgery (NAC-S) is the standard therapy for locally advanced esophageal squamous cell carcinoma (ESCC) in Japan.

**Objective:**

The aim of this phase II trial was to assess the efficacy and safety of the addition of adjuvant S-1 after R0 resection in ESCC patients who received NAC-S.

**Patients and methods:**

Key eligibility criteria included clinical stage IB–III (without T4 disease) ESCC, age 20–75 years, and an Eastern Cooperative Oncology Group (ECOG) performance status of 0 or 1. Patients received adjuvant therapy with four cycles of S-1 (80 mg/m^2^/day) administered orally for 4 weeks of 6-week cycles. The primary endpoint was 3 year relapse-free survival (RFS). If the lower confidence limit for 3 year RFS was >50%, we judged that the primary endpoint of this study was met.

**Results:**

A total of 52 patients were enrolled between January 2016 and January 2019. Two patients were excluded from analysis; five patients were determined to have R1 or R2 resection, and seven patients did not receive adjuvant S-1. The 3-year RFS and overall survival rates in the intention-to-treat population were 72.3% (90% confidence interval [CI] 59.9–81.5) and 85.0% (90% CI 73.9–91.6), indicating that the primary endpoint was met. Grade ≥3 adverse events with an incidence ≥10% included neutropenia (13.2%), anorexia (13.2%), and diarrhea (10.5%). There were no treatment-related deaths.

**Conclusion:**

Adjuvant S-1 after NAC-S showed promising efficacy with a manageable safety profile for patients with resectable ESCC and warrants further evaluation in larger studies.

## Introduction

Esophageal cancer is the seventh most common cancer worldwide,^1^ with the incidence of esophageal adenocarcinoma having increased dramatically in developed countries in recent decades. Most esophageal cancers, including those diagnosed in Japan, are squamous cell carcinomas, with adenocarcinomas accounting for only 2.7% of all esophageal cancers in Japan.^2^

Neoadjuvant chemotherapy and chemoradiotherapy represent standard treatments for locally advanced esophageal cancer regardless of histologic type.^3–6^ The JCOG9907 trial, which compared neoadjuvant cisplatin and fluorouracil (CF) chemotherapy with adjuvant CF chemotherapy for patients with locally advanced esophageal squamous cell carcinoma (ESCC) demonstrated that neoadjuvant chemotherapy improved overall survival (OS) compared with adjuvant chemotherapy (hazard ratio [HR] 0.73, 95% confidence interval [CI] 0.54–0.99).^3^ In a subsequent randomized phase III trial (JCOG1109) conducted in Japan, patients who received neoadjuvant chemotherapy with docetaxel plus CF (DCF) experienced longer survival than patients who received neoadjuvant chemoradiotherapy with CF plus radiotherapy or neoadjuvant CF chemotherapy alone.^4^ Based on these findings, neoadjuvant DCF chemotherapy followed by surgery has been the standard treatment for locally advanced ESCC in Japan since 2022.^5^

In Western countries, the CROSS trial demonstrated longer survival among patients with advanced esophageal cancer, including adenocarcinoma and squamous cell carcinoma, who received neoadjuvant chemoradiotherapy with carboplatin and paclitaxel compared with surgery alone (HR 0.657, 95% CI 0.495–0.871).^6^ In East Asia, the NEOCRTEC5010 trial, in which neoadjuvant chemoradiotherapy was compared with surgery alone in patients with locally advanced ESCC, reported similar results.^7^ Recently, the CheckMate-577 trial confirmed the superiority of adjuvant nivolumab to placebo with respect to disease-free survival (DFS) in patients with locally advanced esophageal cancer with residual pathological disease. Based on the results of CheckMate-577, adjuvant nivolumab is the standard treatment for patients with locally advanced ESCC who received neoadjuvant chemoradiotherapy followed by surgery.^8^ However, the 3-year relapse-free survival (RFS) rate for patients with squamous cell carcinoma in CheckMate-577 was <40%, which was not as favorable as the 64.9% observed in patients with residual pathological disease in JCOG1109. This finding highlights a notable discrepancy between these two studies; therefore, it is not possible to extrapolate the evidence from CheckMate-577 immediately to Japan.

Although perioperative chemotherapy with 5-fluorouracil, leucovorin, oxaliplatin, and docetaxel, referred to as the FLOT regimen, has been established as an accepted standard for esophagogastric adenocarcinoma,^9,10^ perioperative chemotherapy with cytotoxic agents has not yet been established for ESCC.

The same platinum-containing adjuvant chemotherapy regimen is used as neoadjuvant chemotherapy in patients with esophagogastric adenocarcinoma.^9–12^ Four previous randomized trials in locally advanced esophagogastric adenocarcinoma demonstrated a completion rate for all cycles of neoadjuvant chemotherapy of 87–97%, while the completion rate for all cycles of adjuvant chemotherapy was 38–48%. In contrast, among patients who received only adjuvant chemotherapy without neoadjuvant chemotherapy in JCOG9907, 81/108 (75%) patients with esophageal squamous cell carcinoma with lymph node metastases who were scheduled to receive adjuvant chemotherapy completed two cycles of adjuvant chemotherapy. Based on these results, for platinum-containing adjuvant chemotherapy regimens, the completion rate of adjuvant chemotherapy in patients who received neoadjuvant chemotherapy was considered to be insufficient compared with that for patients who did not receive neoadjuvant chemotherapy.

S-1 is an oral anticancer drug consisting of tegafur, 5-chloro-2,4-dihydroxypyridine, and potassium oxonate. S-1 is widely used as adjuvant chemotherapy for gastric cancer (ACTS-GC),^13^ pancreatic cancer (JASPAC01),^14^ biliary tract cancer (ASCOT),^15^ and colorectal cancer (ACTS-CC).^16^ One previous phase II trial in locally advanced esophagogastric adenocarcinoma evaluated the use of three cycles of neoadjuvant triplet chemotherapy consisting of docetaxel, oxaliplatin, and S-1 plus surgery followed by adjuvant S-1 for 1 year.^17^ In this phase II trial, all patients completed all cycles of neoadjuvant chemotherapy, and the completion rate for all cycles of adjuvant chemotherapy was 81%. This high adjuvant chemotherapy completion rate among patients who received neoadjuvant chemotherapy was considered promising.

The aim of this phase II trial, the PIECE trial, was to assess the efficacy and safety of adjuvant S-1 following R0 resection in patients who received neoadjuvant CF chemotherapy. When this trial started accrual in 2015, neoadjuvant CF chemotherapy followed by surgery was the standard treatment in Japan.

## Patients and Methods

### Study Design and Patients

This was a multicenter, open-label, phase II study that recruited patients from 12 academic medical centers in Japan. Inclusion criteria were age 20–75 years, Eastern Cooperative Oncology Group (ECOG) performance status of 0 or 1, adequate organ function (i.e., absolute neutrophil count ≥1500 cells/μL, white blood cell count ≤12,000 cells/μL, platelet count ≥75,000 cells/μL, total bilirubin ≤2.0 mg/dL, aspartate aminotransferase and alanine aminotransferase ≤100 IU/L, serum creatinine ≤1.2 mg/dL, and creatinine clearance ≥50 mL/min), and provision of written informed consent. Patients had clinical stage IB–III (excluding T4) disease based on the 7th Union for International Cancer Control (UICC) TNM classification of histologically confirmed ESCC, adenosquamous cell carcinoma, or basal cell carcinoma. Patients received neoadjuvant chemotherapy consisting of 5-fluorouracil plus cisplatin and were expected to undergo R0 resection by esophagectomy. All patients registered before surgery. The study protocol was approved by the Ethics Committee and the Institutional Review Board of each institution and was conducted in accordance with the ethical principles originating from the Declaration of Helsinki. This trial was registered at the Japan Registry of Clinical Trials with the number jRCTs051180154 (https://jrct.niph.go.jp/latest-detail/jRCTs051180154).

### Procedures

Total or subtotal thoracic esophagectomy, or thoracoscopic esophagectomy, and regional lymphadenectomy were performed after completion of neoadjuvant chemotherapy. Adjuvant chemotherapy was performed within 56 days of surgery. Patients received adjuvant therapy consisting of oral S-1 twice daily for 4 weeks, followed by a 2-week rest period. Three dose levels of S-1 were administered according to body surface area (BSA): <1.25 m^2^, 40 mg twice daily; 1.25 to <1.50 m^2^, 50 mg twice daily; and ≥1.5 m^2^, 60 mg twice daily. Patients with creatinine clearance levels of 50–60 mL/min received lower S-1 doses (BSA <1.25 m^2^, 25 mg twice daily; 1.25 to <1.50 m^2^, 40 mg twice daily; and ≥1.5 m^2^, 50 mg twice daily). Treatment was continued for up to four 24-week cycles. To start each cycle of S-1, patients had to satisfy the following criteria: absolute neutrophil count ≥1200 cells/μL, platelet count ≥75,000 cells/μL, total bilirubin ≤3.0 mg/dL, aspartate aminotransferase and alanine aminotransferase ≤150 IU/L, serum creatinine ≤1.2 mg/dL, and no other non-hematological adverse events grade ≥1 for which an investigator judged administration to be inappropriate.

In addition to the criteria for stopping and restarting S-1 in each cycle, further administration of S-1 in the ongoing cycle was suspended if any of the following adverse events were observed: absolute neutrophil count <1000 cells/μL; platelet count <70,000 cells/μL; grade ≥2 gastrointestinal disorders such as diarrhea, nausea, vomiting, anorexia, or oral mucositis; and no other grade ≥3 non-hematological adverse events. Once administration of S-1 was suspended, the daily dose of S-1 for the next cycle was reduced from 120 mg to 100 mg, from 100 mg to 80 mg, or from 80 mg to 50 mg once daily, depending on BSA, or the administration period was changed from 4 weeks of each 6-week cycle to 2 weeks of each 3-week cycle. All patients were evaluated by computed tomography every 6 months until recurrence or withdrawal of consent. Adverse events were evaluated according to the National Cancer Institute Common Terminology Criteria for Adverse Events version 4.0.

### Statistical Analysis

All survival analyses were conducted for all eligible patients and all R0-resected patients. Treatment delivery and safety were assessed in all treated patients. The primary endpoint was 3-year RFS rate, and secondary endpoints were OS, RFS, treatment completion rate, treatment continuation rate per time point, incidence of adverse events, and incidence of treatment-related deaths. OS was defined from the date of esophagectomy to the date of death due to any cause, censored as of the last date the patient was documented to be alive. RFS was defined from the date of esophagectomy until relapse or death from other causes, censored as of the last date the patient was documented to be alive without any evidence of relapse. Incomplete resection was not regarded as an event or censoring due to no relapse. Time-to-event distributions were estimated using the Kaplan–Meier method, and CIs were calculated using Greenwood’s formula. For comparisons of patient subgroups, a univariate Cox proportional hazards model was used. To explore prognostic factors in subgroups, analyses were performed according to sex, age (<65 years vs. ≥65 years), performance status (0 vs. 1), surgical method (open transthoracic esophagectomy vs. thoracoscopic esophagectomy), cT (cT1-2 vs. cT3), cN (cN0 vs. cN1-3), and tumor location (middle thoracic vs. upper thoracic vs. lower thoracic). Statistical analysis was performed using STATA version 17 (StataCorp LLP, College Station, TX, USA).

A minimum sample size of 43 patients with R0 resection was required to provide a power of 0.80 with a one-sided significance level of 0.10, and to detect an alternative 3-year RFS rate of 66% compared with a null hypothesis of 50% on the binomial distribution, according to calculations using PASS software (PASS 11; NCSS, Kaysville, UT, USA). The 3-year PFS rate from the date of surgery in 152 patients who received at least one course of neoadjuvant chemotherapy followed by surgery was 51.2% based on available data from JCOG9907 (unpublished data), therefore we set the threshold at 50%. A total of 50 patients who were expected to undergo R0 resection by esophagectomy was planned for enrolment, with some withdrawals due to R1–2 resection.

## Results

### Patient Characteristics

**Fig. 1:**
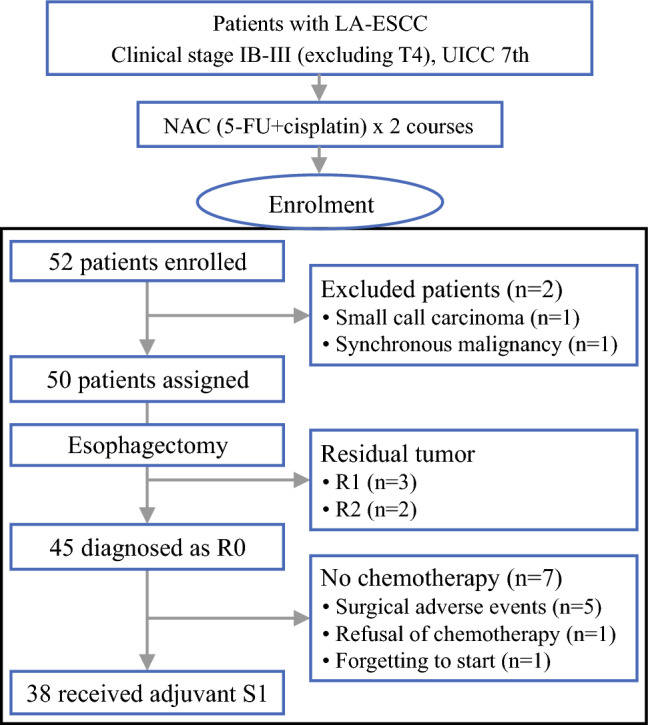
PIECE trial flowchart. *LA-ESCC* locally advanced esophageal squamous cell carcinoma, *NAC* neoadjuvant chemotherapy, *UICC* Union for International Cancer Control, *5-FU* 5-fluorouracil

Between January 2016 and January 2019, 52 patients were recruited from 12 institutions. The trial scheme and flow diagram are shown in Fig. 1. Overall, 2 of the 52 patients were ineligible due to ineligible histologic type (small cell carcinoma) and synchronous malignancy. Patient characteristics are summarized in Table 1. Of the 50 eligible patients, 45 received two cycles of neoadjuvant chemotherapy. Of the 5 patients who received only one cycle of neoadjuvant chemotherapy, 4 were due to adverse events related to neoadjuvant chemotherapy and 1 was due to progressive disease. The median follow-up period of censored patients (clinical data cut-off date 18 April 2022) was 4.5 years (range 0.2–5.7 years).

**Table 1:** Patient characteristics

		*N* = 50
Age, years	Median	62
	Range	40–72
	<65	29
	≥65	21
Sex	Male	38
	Female	12
ECOG PS at registration	0	46
	1	4
Tumor location	Upper thoracic	5
	Middle thoracic	22
	Lower thoracic	23
cT stage	cT1	10
	cT2	10
	cT3	30
cN stage	cN0	12
	cN1	27
	cN2	11
	cN3	0
cStage	IB	6
	II	17
	III	27
No. of neoadjuvant chemotherapy cycles	1	5
	2	45
Reason for discontinuation of neoadjuvant chemotherapy	Adverse events	4
	Disease progression	1

*ECOG PS* Eastern Cooperative Oncology Group performance status

### Treatment Disposition

Surgical intervention was performed in 50 eligible patients, with R0 resection achieved in 45 patients. Of the eligible patients, 45 underwent thoracoscopic esophagectomy and 39 underwent laparoscopic gastric mobilization. All eligible patients underwent D2 or higher lymphadenectomy, and 18 patients underwent D3 lymphadenectomy. The median number of lymph node dissections was 52 (range 28–115). All patients with ypM1 disease had supraclavicular lymph node metastases and none had distant organ metastases. The complete pathological response rate to neoadjuvant chemotherapy was 4.0%. Surgical outcomes are summarized in Table 2.

**Table 2:** Surgical outcomes

		*N* = 50
Thoracic approach	Open	5
	Thoracoscopy	45
Abdominal approach	Open	11
	Laparoscopy	39
Reconstruction route	Retrosternal	13
	Posterior mediastinal	37
Resection margin	R0 resection	45
	R1 resection	3
	R2 resection	2
ypT stage	ypT0	2
	ypTis	1
	ypT1	15
	ypT2	8
	ypT3	21
	ypT4	3
ypN stage	ypN0	23
	ypN1	15
	ypN2	9
	ypN3	3
ypM stage	ypM0	46
	ypM1	4
ypStage	0	3
	I	11
	II	15
	III	17
	IV	4
Histologic response of primary site^a^	Grade 0 (ineffective)	4
	Grade 1a (slightly effective a)	38
	Grade 1b (slightly effective b)	1
	Grade 2 (moderately effective)	5
	Grade 3 (no residual tumor)	2
Extent of lymphadenectomy	D2 resection	32
	D3 resection	18

^a^Japanese Classification of Esophageal Cancer 11th Edition

Overall, 7 of the 45 patients did not receive S-1. Reasons for not receiving S-1 were postoperative adverse events in 5 patients, refusal of chemotherapy in 1 patient, and ‘other’ in 1 patient. As a result, 38 patients received at least one dose of S-1. All 38 treated patients were included in the toxicity analysis.

Of 38 patients who received S-1, 32 patients completed the protocol treatment. The remaining 6 patients discontinued treatment due to adverse events related to S-1. The median relative dose intensity among all patients was 85.8% (interquartile range [IQR] 73.1–93.8%) [Table 3].

**Table 3:** Adherence to adjuvant S-1

		1 course [*n* = 38]	2 courses [*n* = 35]	3 courses [*n* = 34]	4 courses [*n* = 32]
ECOG PS	0	33	29	27	26
	1	5	6	7	6
Treatment schedule	6-week cycle	30	19	18	16
	3-week cycle	8	16	16	16
Relative dose intensity for each cycle (%)	Median	96.4	89.6	83.3	80.3
	IQR	78.6–100	75.0–100	75.0–100	50.0–100
Relative dose intensity for all cycles (%)	Median	85.8			
	IQR	73.1–93.8			

*ECOG PS* Eastern Cooperative Oncology Group performance status, *IQR* interquartile range

### Survival Outcomes

**Fig. 2:**
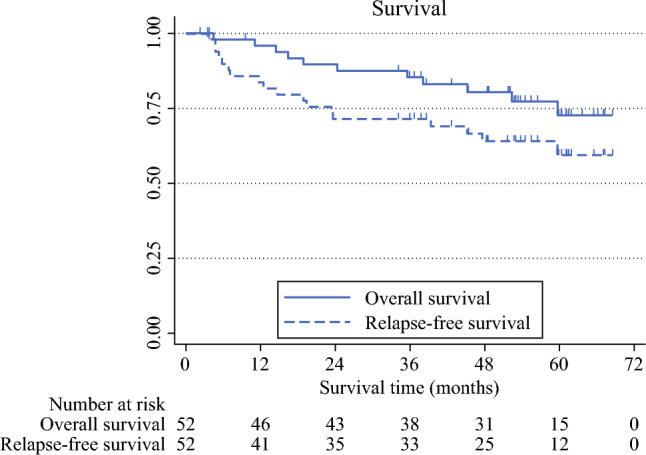
Kaplan–Meier curves for overall survival and relapse-free survival

The 3-year RFS and OS rates were 72.3% (90% CI 59.9–81.5%) and 85.0% (90% CI 73.9–91.6%), respectively, among eligible patients (Fig. 2). Subgroup analyses using a univariate Cox proportional hazards model identified ypT3-4 stage for RFS and open esophagectomy for OS as poor prognostic factors (Table 4).

**Table 4:** Subgroup analyses of relapse-free and overall survival

		*N *= 50	3-year RFS rate (90% CI)	HR (90% CI)	*p*-Value		3-year OS rate (90% CI)	HR (90% CI)	*p*-Value
Age, years	<65	29	65.4 (47.9–78.3)	Reference			80.7 (64.0–90.2)	Reference	
	≥65	21	80.9 (61.7–91.1)	0.61 (0.26–1.42)	0.345		90.4 (72.5–96.9)	0.98 (0.36–2.68)	0.986
Sex	Male	38	71.0 (56.8–81.2)	Reference			84.2 (71.4–91.6)	Reference	
	Female	12	77.7 (44.6–92.4)	0.79 (0.27–2.26)	0.718		88.8 (54.3–97.7)	0.82 (0.22–2.98)	0.805
PS at registration	0	46	74.4 (61.5–83.5)	Reference			85.9 (74.3–92.5)	Reference	
	1	4	50.0 (10.3–80.9)	2.41 (0.69–8.41)	0.247		75.0 (22.3–94.6)	1.25 (0.22–7.04)	0.832
Tumor location	Upper thoracic	5	75.0 (22.3–94.6)	0.43 (0.07–2.42)	0.423		100 (–)	–	1
	Middle thoracic	22	63.6 (44.3–77.7)	Reference			77.2 (58.3–88.4)	Reference	
	Lower thoracic	23	80.9 (61.7–91.1)	0.53 (0.22–1.25)	0.227		90.4 (72.5–96.9)	0.52 (0.18–1.47)	0.305
cT stage	cT1-2	20	83.3 (62.3–93.2)	Reference			94.4 (74.3–98.9)	Reference	
	cT3	30	65.5 (48.9–77.8)	1.06 (0.85–5.02)	0.178		79.1 (63.2–88.7)	2.21 (0.71–6.80)	0.246
cN stage	cN0	12	63.6 (35.4–82.1)	Reference			72.7 (43.7–88.4)	Reference	
	cN1-3	38	75.0 (60.7–84.7)	0.69 (0.29–1.67)	0.498		88.8 (76.4–94.9)	0.50 (0.18–1.42)	0.282
Thoracic approach	Open	5	60.0 (19.1–85.4)	Reference			80.0 (31.3–95.8)	Reference	
	Thoracoscopic	45	73.8 (60.7–83.1)	0.49 (0.17–1.41)	0.27		85.6 (73.8–92.4)	0.26 (0.08–0.80)	0.049
ypT stage	ypT0-2	26	87.5 (70.7–94.9)	Reference			91.4 (75.1–97.2)	Reference	
	ypT3-4	24	56.5 (38.0–71.4)	3.45 (1.43–8.31)	0.02		78.2 (59.9–88.9)	2.16 (0.77–6.09)	0.218
ypN stage	ypN0	23	86.3 (68.4–94.4)	Reference			95.4 (78.5–99.1)	Reference	
	ypN1-3	27	60.1 (42.2–74.0)	2.08 (0.89–4.83)	0.151		76.0 (58.3–86.9)	1.82 (0.64–5.14)	0.339

*RFS* recurrence-free survival, *CI* confidence interval, *HR* hazard ratio, *OS* overall survival, *PS* performance status

Among the 45 patients in whom R0 resection was achieved, 3 experienced a pathological complete response (pCR), while the remaining 42 did not. In a post hoc analysis, the 3-year RFS and OS rates were 79.4% (90% CI 66.2–87.9%) and 92.2% (90% CI 81.1–96.9%), respectively, in the 42 patients without pCR.

Among the 16 observed events of disease recurrence, 6 patients (12%) had locoregional recurrence and 10 patients (20%) had distant recurrence. A total of 15 patients received subsequent therapy, including chemoradiotherapy (*n* = 8), chemotherapy (*n* = 6), and radiotherapy alone (*n* = 1). Of the 11 observed deaths, 10 patients (20%) died from primary disease and 1 patient (2%) died suddenly without cancer recurrence.

### Safety Related to S-1

Common adverse events (i.e. frequency ≥40%) of any grade were decreased white blood cell counts, decreased neutrophil counts, anemia, hypoalbuminemia, increased aspartate aminotransferase, fatigue, and anorexia (Table 5). The most common grade ≥3 adverse events were decreased neutrophil count in 5 patients (13.2%), anorexia in 5 patients (13.2%), and diarrhea in 4 patients (10.5%). Two patients experienced grade 4 adverse events, i.e. hypokalemia and ileus. No treatment-related deaths occurred.

**Table 5:** Adverse events

Event, *n*	Grade 1	Grade 2	Grade 3	Grade 4	Grade ≥3, %
White blood cell decreased	3	15	0	0	0
Neutrophil count decreased	10	10	5	0	13.2
Anemia	23	9	3	0	7.9
Platelet count decreased	13	1	0	0	0
Hypoalbuminemia	27	5	1	0	2.6
Aspartate aminotransferase increased	20	1	0	0	0
Alanine aminotransferase increased	14	0	0	0	0
Blood bilirubin increased	5	1	0	0	0
Creatinine increased	6	0	0	0	0
Hyponatremia	12	0	1	0	2.6
Hypokalemia	9	0	1	1	5.3
Hyperkalemia	1	0	0	0	0
Fever	4	1	0	0	0
Fatigue	9	6	1	0	2.6
Diarrhea	4	6	4	0	10.5
Nausea	12	1	2	0	5.3
Vomiting	5	1	0	0	0
Mucositis oral	5	1	0	0	0
Skin hyperpigmentation	4	0	0	0	0
Palmar-plantar erythrodysesthesia syndrome	2	3	0	0	0
Rash maculopapular	7	2	0	0	0
Anorexia	17	4	5	0	13.2
Pharyngitis	1	1	0	0	0
Watering eyes	9	1	0	0	0
Abdominal pain	1	1	1	0	2.6
Epistaxis	5	0	0	0	0
Peripheral sensory neuropathy	1	0	0	0	0
Dysphagia	0	1	0	0	0
Cough	2	0	0	0	0
Upper respiratory infection	0	1	0	0	0
Lung infection	0	1	1	0	2.6
Aspiration	0	1	1	0	2.6
Pruritus	0	1	0	0	0
Constipation	1	0	0	0	0
Rash acneiform	0	1	0	0	0
Flu-like symptoms	0	0	1	0	2.6
Upper gastrointestinal hemorrhage	1	0	0	0	0
Dysgeusia	1	0	0	0	0
Pneumonitis	0	1	0	0	0
Papulopustular rash	0	1	0	0	0
Gastroesophageal reflux disease	0	0	1	0	2.6
Ileus	0	0	0	1	2.6

## Discussion

The results of the PIECE trial yielded promising outcomes, with a 3-year RFS rate of 72.3% and a 3-year OS rate of 85.0% among eligible patients; the trial met its primary endpoint.

Neoadjuvant CF chemotherapy followed by surgery in JCOG9907 resulted in 3-year RFS and OS rates of 49.2% and 62.7%, respectively.^3^ JCOG9907 was conducted before the immune checkpoint inhibitor era, over 10 years ago. In a subsequent randomized phase III trial (JCOG1109) conducted from 2012 to 2018, neoadjuvant CF chemotherapy followed by surgery resulted in a 3-year RFS rate of 48.1% and a 3-year OS rate of 62.7%.^4^ The PIECE trial differs slightly from JCOG1109 with respect to subjects and surgical procedures. In the PIECE trial, 59.6% of patients had clinical T3 disease, compared with 68.8% in the neoadjuvant CF group of JCOG1109. In addition, in the PIECE trial, 90.0% of patients underwent thoracoscopic esophagectomy, compared with 50.5% in the neoadjuvant CF group of JCOG1109. Recently, in a randomized phase III trial (JCOG1409) comparing thoracoscopic esophagectomy with open esophagectomy for thoracic esophageal cancer, the second planned interim analysis demonstrated non-inferiority of thoracoscopic esophagectomy to open esophagectomy with respect to OS, and the 3-year OS rate in the thoracoscopic esophagectomy group was better than that in the open esophagectomy group (82.0% vs. 70.9%), with an HR of 0.64 (95% CI 0.39–1.06).^18^ Based on these results, it is possible that the improved survival in the PIECE trial was due not only to the efficacy of adjuvant S-1 but also to the influence of thoracoscopic esophagectomy. However, the 3-year RFS and OS rates for patients in the thoracoscopic esophagectomy group with the same stage disease (clinical stage IA–III) as JCOG1409 (accrual period 2015–2022), which was conducted at the same time as the PIECE trial, were 61.8% and 74.0%, respectively, and 55.9% of patients had clinical T3 disease. Collectively, these results suggest that the addition of adjuvant S-1 in patients treated with neoadjuvant CF chemotherapy followed by surgery may be effective, although there are limitations because the comparisons were made between different clinical trials conducted at the same time.

In previous clinical trials, the adjuvant platinum-containing chemotherapy completion rate in patients who received neoadjuvant chemotherapy was considered to be insufficient compared with that for patients who did not receive neoadjuvant chemotherapy.^3,9–12^ In a previous phase II trial in locally advanced esophagogastric adenocarcinoma, all patients completed all neoadjuvant chemotherapy cycles, and the completion rate for all cycles of adjuvant chemotherapy was 81%.^17^ Based on these results, adjuvant S-1 was considered promising and thus was used in the PIECE trial. The 6-month treatment completion rates for adjuvant S-1 were 78% in ACTS-GC,^13^ 72% in JASPAC01,^14^ 72% in ASCOT,^15^ and 77% in ACTS-CC.^16^ These rates are similar to those observed in the PIECE trial; however, it is important to note that the patients in these previous trials did not receive neoadjuvant chemotherapy. Nevertheless, adjuvant S-1 was expected to have a high completion rate regardless of the presence or absence of neoadjuvant chemotherapy.

As a result of CheckMate-577, adjuvant nivolumab is the standard treatment for locally advanced ESCC patients treated with neoadjuvant chemoradiotherapy followed by surgery.^8^ CheckMate-577 confirmed the superiority of adjuvant nivolumab to placebo with respect to DFS in patients with locally advanced esophageal cancer with residual pathological disease who have a high risk of recurrence and a poor prognosis.^19^ An important issue with adjuvant nivolumab is the chronic and often persistent immune-related adverse events observed after the end of treatment.^20–22^ In contrast, such events have not been reported with adjuvant S-1,^13–16^ including in the PIECE trial. Another important issue with adjuvant nivolumab is the cost of 1 year of treatment, which is about 70 times higher than that of 6 months of S-1 in Japan. However, although S-1 is also promising with respect to late toxicity and cost, it has been suggested that the pharmacokinetics and toxicity of S-1 differ in European and North American patients, particularly with regard to the occurrence of diarrhea, which may require dose adjustment.^23,24^

The 3-year RFS rate for squamous cell carcinoma patients in the CheckMate-577 trial was <40%, which was not as favorable as the 64.9% observed in patients with residual pathological disease in JCOG1109. This finding highlights a notable discrepancy between the two studies; therefore, it is not possible to extrapolate the evidence of the CheckMate-577 trial immediately to Japan.

Based on these results, we are conducting a phase III trial (JCOG2206) to confirm the superiority of the addition of adjuvant therapy with nivolumab or adjuvant chemotherapy with S-1 to the standard treatment, neoadjuvant chemotherapy with DCF or CF followed by curative esophagectomy for patients with locally advanced ESCC with no pCR.^25^ JCOG2206 was registered at the Japan Registry of Clinical Trials with the number jRCTs031230219 (https://jrct.niph.go.jp/latest-detail/jRCTs031230219).

The present study was limited by the small sample size and the lack of a control group. In the PIECE trial, neoadjuvant chemotherapy consisted of CF without concurrent radiotherapy or without docetaxel, and the timing of enrolment differs from that of JCOG1109 and CheckMate-577. The standard neoadjuvant treatment became neoadjuvant DCF as a result of JCOG1109 in Japan.

## Conclusion

Adjuvant S-1 showed promising efficacy with a manageable safety profile in patients with resectable ESCC after neoadjuvant chemotherapy followed by surgery and warrants further evaluation in larger studies, including JCOG2206.

## Data Availability

The datasets generated and/or analyzed during the current study are available from the corresponding author on reasonable request. The data are not publicly available due to restrictions on the inclusion of information that could compromise the privacy of research participants.
